# Consumer Engagement in Mobile Application (App) Interventions Focused on Supporting Infant Feeding Practices for Early Prevention of Childhood Obesity

**DOI:** 10.3389/fpubh.2019.00060

**Published:** 2019-03-22

**Authors:** Sarah Taki, Catherine G. Russell, Li M. Wen, Rachel A. Laws, Karen Campbell, Huilan Xu, Elizabeth Denney-Wilson

**Affiliations:** ^1^Health Promotion Unit, Camperdown, NSW, Australia; ^2^Sydney School of Public Health, Sydney Medical School, University of Sydney, Camperdown, NSW, Australia; ^3^Centre for Obesity Management and Prevention Research Excellence in Primary Health Care, Sydney, NSW, Australia; ^4^Centre of Research Excellence, Early Prevention of Obesity in Childhood, Sydney, NSW, Australia; ^5^Centre for Advanced Sensory Science, School of Exercise and Nutrition Sciences, Deakin University, Geelong, VIC, Australia; ^6^School of Exercise and Nutrition Sciences, Institute for Physical Activity and Nutrition, Deakin University, Geelong, VIC, Australia; ^7^Sydney Nursing School, University of Sydney, Camperdown, NSW, Australia

**Keywords:** mHealth, smartphone, obesity, infant, children, parents, nutritional requirements

## Abstract

**Background and Aims:** There has been increasing interest in using mobile applications (“apps”) for innovative health service delivery and public health interventions. This paper describes two independent studies investigating mothers' or pregnant women's perceptions of, interest in and experiences with technological devices, apps and websites about infant feeding practices.

**Methods:** Study 1 was a cross-sectional survey conducted with 107 pregnant women in their third trimester in late 2016 and early 2017. Multiple logistic regression analyses were conducted to examine factors associated with their app usage. The second was a qualitative study of 29 mothers of infants aged <1 year conducted in 2014. Thematic network analysis was used to explore the themes from the transcribed interviews.

**Results:** Study 1 found that the use of apps was common among the pregnant women, with 100% having previously downloaded an app on their phone either free or paid. About 60% had used an app for health purposes. The majority reported that they were likely to use an app promoting healthy infant feeding practices, including 30% extremely likely and 53% very likely. Women with university or other tertiary level of education were more likely to use an app for promoting healthy infant feeding practices than those with other levels of education (adjusted odds ratio 3.22, 95% confidence interval 1.28–8.13). The qualitative interviews found that all the mothers were interested in a mobile program to support them with infant feeding practices. Participants felt they would benefit from individualized messages although did not want them to be sent too frequently. Further, participants also expressed the importance of having non-judgmental information and they were interested in receiving information using different modes such as videos, SMS or an app.

**Conclusions:** Both studies suggest that using apps for promoting healthy infant feeding practices is acceptable from the perspective of mothers. There is great potential for health promotion practitioners to be engaged in app development for the purpose of promoting health in early years and health promotion in general.

## Introduction

The transition to parenthood requires the acquisition of broad ranging knowledge to cope with the demands of caring for a newborn (1, 2). A key part of this relates to infant feeding. Key decisions about whether to breastfeed (and for how long), to formula feed and the age at which to introduce solids, can be influenced by a number of factors, including mothers' knowledge, skills and support as well as their motivations (3–5). Maternal decisions about which infant feeding practices to use are critical to infants' health, as they can influence weight gain, in particular rapid weight gain, which is a major contributor to obesity risk in both early and later life (6, 7). Providing mothers with access to reliable and evidence-based information about infant feeding is therefore crucial due to links between these practices and the development of infants' weight status, healthy eating behaviors and food preferences (6, 8).

Mothers obtain infant feeding information from multiple sources including books, the internet, friends and family, and from health practitioners (3, 9). With the rapid expansion of technology, mothers are now turning to digital sources for information about pregnancy, birth and infant care (10, 11). An Australian study which included surveying a total of 410 pregnant women and mothers (87%), identified that almost three quarters used a pregnancy app and less than half (43%) used parenting apps (11).These platforms are beneficial in providing social support to this demographic, especially where they may be socially isolated, time poor and reliant on reassurance in caring for their infant (12–14).

There has been increasing interest in using mobile applications (“apps”) for innovative, cost-effective and sustainable health service delivery and public health interventions for supporting mothers during pregnancy and the postpartum period (15, 16). Currently there are over 100,000 “health apps” available internationally with approximately 3.7 billion downloads in 2017 (17). While “health apps” are numerous, several reviews have suggested that most consumer apps more broadly are not developed by credible sources, and they generally do not align with the clinical guidelines for the targeted health topic (18–21). Consistent with this, a systematic analysis conducted to explore the quality of infant feeding websites and apps identified that very few were developed by credible sources or provided accurate information that aligned with the Australian Infant Feeding Guidelines (22, 23).

Smartphone health apps offer a variety of features including tracking health behaviors, sending “nudges” for health behaviors through push notifications, music features, pictures and video features as well as providing health information (24–26). Previous studies have been conducted on women's use of parenting apps (11, 27, 28). However, there are few studies which have explored the use of apps and features that would engage mothers to use an app that would support them with infant feeding. Including end users in the development of apps has become commonplace and is considered to be an important process for engagement in health programs delivered through technology. For example a number of studies focused on app development in adult populations have provided important guidance features they would find beneficial and use on a health smartphone app (29, 30). Developing health apps with end users and amongst health professionals and researchers is considered as a best practice approach (29, 30).

The aims of this paper are to report on the findings of two studies that investigated pregnant women and new mothers' engagement and interest in using apps to promote healthy infant feeding behaviors. Another aim was to also explore mothers' current access and use of technological devices, apps and websites for information on infant care.

## Methods

### Study 1: A Cross-Sectional Survey

The survey was conducted at baseline with women participating in a feasibility study of the Healthy Beginnings App in late 2016 and early 2017. Pregnant women who attended antenatal clinics in one of the largest teaching hospital located in the inner west Sydney, Australia were approached by two research assistants with a letter of invitation and information about the study. Women were eligible to participate if they were in their third trimester, aged 18 years and over, were able to communicate in English, lived in the local areas and used an iPhone (note: the Healthy Beginnings App was currently only available from the iPhone app store). Once eligibility was established and consent obtained, women were asked to complete a survey with a research assistant. The study was approved by the Sydney Local Health District Research Ethics and Governance Office (HREC/16RPAH/280).

Table 1 presents the survey questions, which included basic demographics and app usage (such as app download, and its purpose) as well as likelihood of using an app for promoting infant feeding practices. The questions were developed and pilot tested by the project team. The statistical analyses were conducted using Stata 13. Descriptive analysis was conducted to summarize participants' demographic characteristics and their app usage. Multiple logistic regression analyses were conducted to examine whether demographics (such as age, employment status and educational level) were associated with their app usage.

**Table 1:** Study 1: survey questions used in the cross-sectional survey.

Have you ever downloaded apps for your device? □ Yes - Always Free □ Yes - Always Paid □ Yes - Free and Paid □ No Have you ever used an App for health information purpose? □ Yes □ No How likely would you be to use the App for promoting healthy infant feeding practices if it is available on your device? □ It already is available on my device □ Extremely likely □ Very likely □ Moderately likely □ Slightly likely □ Not at all likely **[Demographics]** What is your date of birth? _______ / _______ / _______ day month year □ Refused [*don'tread*] In which country were you born? □ Australia □ Other, please specify _______________________ □ Refused [*don'tread*] What language do you usually speak at home? □ English □ Other, please specify _______________________ □ Refused [*don'tread*] How would you describe your current employment status? (*If more than one, tick the option **highest** on the list)* □ Employed full-time (include self-employed) □ Employed part-time (include self-employed) □ Paid maternity leave – employed □ Unpaid maternity leave – employed □ Unemployed □ Home duties □ Student and working □ Student and not working □ Retired □ Full-time carer □ Unable to work due to health problems □ Casually employed □ Refused [*don'tread*] What is your present marital status? □ Married (refers to registered marriages) □ De-facto partner □ Divorced □ Separated but not divorced □ Widowed □ Never married □ Refused [*don'tread*] What is the highest qualification you have completed? □ Completed primary school □ Completed years 7 to 9 □ Completed School Certificate or Intermediate Certificate or Year 10 or 4th Form □ Completed High School Certificate or Leaving Certificate or Year 12 or 6th Form □ TAFE certificate or diploma □ University or some other tertiary institute degree or higher □ Other, please specify ________________________________________ □ Don't know [*don't read*] □ Refused [*don'tread*]

### Study 2: Qualitative Interviews

This qualitative study was adopted to inform the development of an mHealth intervention on healthy infant feeding practices, the Growing Healthy feasibility trial (31). The study aimed to investigate mothers' interest in the development of a website and smartphone app that provides infant feeding information. This study also explored mothers' infant feeding beliefs and behaviors, although these findings are published elsewhere (5).

#### Participants

This study was advertised on a banner on the monthly Playgroups NSW e-newsletter between January and March 2014. Playgroups NSW offered playgroups to parents and carers with children aged 0–5 years and had approximately 25,000 members. The banner which advertised the study included a link to a web-based survey (SurveyMonkey®) where mothers were requested to provide demographic information and contact details to determine eligibility. Mothers were eligible if they did not have a university degree (considered a proxy for lower socioeconomic position) (32) were the primary caregiver, were fluent in English, had a child who was younger than 1 year, with no major health problems. Eligible participants were emailed a plain language statement and a consent form. Verbal consent was provided prior to conducting the one-on-one telephone interview. Interviews were conducted until data saturation was reached.

#### Interviews

The interview schedule was developed from components measured in a developed app quality tool (22) and also engagement strategies previously used in other mHealth programs (25, 26, 33).

The interview schedule covered the following topics:

- Current access to a mHealth device (mobile phone, computer, laptop, iPad, notepad)
- Current usage of the internet
- Preferences for or interest in a mHealth program
- Feedback on features that may be of interest in a mHealth program to support with infant feeding (smartphone application, messages (including frequency of messages), forums, sharing information with other carers, recipes and videos on infant feeding)

The interview was pilot tested with five mothers who met the eligibility criteria, and refinements were made to improve the clarity and flow. The interviews were conducted by two researchers (ST and LA) and audio-recorded with participants' permission. Mothers were offered an AUD30 supermarket voucher to compensate them for their time. The University of Technology Sydney Human Research Ethics Committee (ID 2013000463) granted ethics approval to conduct this study.

#### Analysis

The audio-recorded interviews were transcribed verbatim, and the audio interviews checked against the transcripts to ensure accuracy. Data were imported into NVivo 10® a qualitative data analysis software package to code, store, sort and retrieve results from de-identified transcripts. Authors ST and CGR independently developed coding manuals using Attride-Stirling thematic analysis network (34) to guide the development. Two iterations took place in developing the manual with the researchers coding 5 transcripts each to identify themes and relevant quotes. Sub-themes and broader themes were developed for the coding manual. The coding manual was discussed after each iteration and discrepancies in the coding manual and codes were resolved through discussion. The inter-rater reliability of the data was measured using the Coding Comparison query on NVivo 10®. This function calculates the percentage agreement of coding between the two researchers. Each theme is then given a Kappa Coefficient score, where Kappa >0.75 indicates “excellent” agreement, Kappa 0.40 < 0.75 is “fair to good” and Kappa < 0.40 is “poor” agreement (35).

## Results

### Study 1: A Cross-Sectional Survey

A total of 107 pregnant women completed the survey out of 186 approached. Among them, 31% were aged <30 years, 43% were aged between 30 and 34 years and 26% were aged older than 34 years. Forty-five per cent were born outside Australia and 65% were employed including 13% on paid and unpaid maternity leave. Sixty per cent had university or other tertiary education. The use of apps was common with 100% having downloaded an app on their phone either free or paid. About 60% had used an app for health information purposes. The majority were either extremely likely (30%) or very likely (53%) to use an app that promoted healthy infant feeding practices if it was available. Table 2 shows sample characteristics.

**Table 2:** Study 1: participant characteristics.

**Variables**	***n* (%)**
**AGE**	
< 30	33 (31)
31–34	46 (43)
≥35	28 (26)
**COUNTRY OF BIRTH**	
Australia	59 (55)
Overseas	458 (45)
**LANGUAGE SPOKEN AT HOME**	
English	78 (73)
Other	29 (27)
**EMPLOYMENT**	
Employed	70 (65)
Other	37 (35)
**EDUCATION**	
University or higher	62 (58)
Other	45 (42)
**MARITAL STATUS**	
Married	79 (76)
De-factor partner	24 (23)
Never married	1 (1)

Using multiple logistic regression analyses, level of education was the only factor that was significantly associated with “extremely likelihood” of participants using an app for promoting healthy infant feeding practices. Mothers with university or other tertiary level of education were more likely to use an app for promoting healthy infant feeding practices than those with other levels of education (adjusted odds ratio 3.22, 95% confidence interval 1.28–8.13). Other demographic factors were not found to be associated with “extremely likelihood” of using an app for promoting healthy infant feeding practices.

### Study 2: Qualitative Interviews

A total of 120 mothers expressed an interest to participate in the qualitative interviews through a Survey Monkey link that included the eligibility form. Of these 29 mothers were eligible and were interviewed between February and March 2014.The other 91 women were excluded from the study either due to not meeting the inclusion criteria or no longer wanted to participate after being contacted. The participants were recruited from New South Wales (*n* = 17) and the Australian Capital Territory (*n* = 12) in Australia. Table 3 illustrates participant characteristics. Participants were aged 21–38 years, majority had completed trade certificates (*n* = 17) and were first time mothers (*n* = 15). The infants were aged from 2 to 11 months (mean = 6.5 months) and included 13 females and 16 males. The mean duration of the interviews was 43 min and ranged from 23 to 78 min. Inter-rater reliability for the coded data on the mothers “*technology usage”* and “*interest in a mHealth program”* was rated as “almost perfect agreement” (Kappa = 0.88) (35).

**Table 3:** Study 2: participant characteristics.

**Variables**	***n***
**AGE**	
≤30	18
31–34	6
≥35	5
**CULTURAL BACKGROUND**	
Australia	25
Overseas	4
**EDUCATION LEVEL**	
TAFE	18
Completed year 12 or lower)	11
**PARITY**	
Primiparous	26
**INFANT AGE**	
Range	2–44 weeks
Mean	25.7 weeks

### Exploration of Mothers' Interest in an mHealth Intervention

Overall majority of the mothers felt that an mHealth program and offering various features to support them with infant feeding practices is beneficial.

#### Technology Usage

##### Access to the internet

Mobile phones was the most commonly reported device that mothers used to access the internet, with Samsung and iPhone as the most commonly owned brands. In particular mobile phones were thought to be more practical compared to computers or laptops and used while caring for their child *[I probably use my internet phone a lot more … I'll just let her sleep in my arms, and I'll jump on my phone. Mother 24]*.

##### Sources accessed on the internet

All of the mothers reported using the internet and majority have used it to search for various websites that provide infant feeding information. Google however, appeared to be the “go to” source *[I'll type my question in to Google and whatever comes up and I usually use five websites…sometimes none of them are really true…just my common sense if I think it sounds like it would work or I'll give it a go. Mother 28]*. Mothers group forums or blogs were also used to find solutions for questions they had on infant feeding *[when we were having difficulties, it always seemed to be in the middle of the night when you felt like you have no support…that covers partly why the Facebook page because there's always someone up feeding a baby or trying to settle a baby for you to talk to. Mother* 4*]*.

Very few mothers reported using apps to support with infant feeding information. Some of the apps that mothers reported using were for tracking the baby's weight and timing the frequency of feeds *[I was using an app when I was tracking feeding but I've stopped completely for a while. Mother* 21] and also to help with settling techniques *[Wonder Weeks…helped me when he was really fussy, because he was really unsettled. Mother* 28*]*.

### Engagement in an mHealth Program

Using mHealth such as an app, website or receiving SMSs to provide them with infant feeding information was an acceptable method by all of the mothers interviewed. Despite this being said, there were some concerns regarding the usefulness of such a program for some individuals. Reasons for this varied including multiparous mothers who felt they had sufficient experience with infant feeding *[Not for me, but I definitely would think a lot of first time mums in particular would. Mother* 7*]*. A lack of time to access the app was another reason *[Depending if I had time to read it or not. Mother* 22*]* and whether it fulfilled their needs around infant feeding advice [*it can be helpful, but you end up just going on your own, what your own needs are for the day, what you've got covered, what you feel like giving your baby. Mother 8]*.

#### Interest in Features of an mHealth Program

##### Frequency of messages

All of the mothers interviewed were interested in the feature of receiving personalized messages related to their baby's age and their feeding method. Majority of the mothers suggested that weekly messages would be appropriate particularly because babies grow rapidly within the first year of life. Further it was emphasized by a number of mothers that having too frequent messages would not be ideal and may potentially lead to disengagement *[I get frequent SMSs'…I sort of tend to just delete them, whereas if it's once or twice a week with some really good information, I would take more time to stop and look at it. Mother* 7*]*.

##### Videos

Video messages were seen as a learning aid, in particularly if they were based on the preparation of baby food *[Yes…I'm not a great cook so if I knew how to do things it would be good. Mother 13]*. However, a couple of mothers believed that these would be useful according to the mothers' experience [*I mean, that's good for first time mums definitely…when you get thrown in the deep end you're like “What do I do? Mother 3]*.

##### Interconnectivity

More than half of the participants believed that sharing information from the mHealth program would be a beneficial feature if the information was reliable *[Yes, that would be good to shove in new parents' faces and go no that's not right. Mother 14]* and also to provide knowledge to other carers *[Well I think it would be a good idea, because sometimes grandparents…a bit of an old fashioned view on what to do and how to feed the baby kind of thing. Mother 2]*. Although, some did not feel that it was useful as they thought other carers would not bother looking at the information shared *[Not really. I mean, my husband – he just wouldn't look at it anyway, to be honest. He just goes with what I say. Mother 8*].

##### Infant Weight Tracking

Tracking the infants' weight with an app was one of the least favored features that mothers wanted to see in an app. In particular, they felt that getting their child's weight tracked by health professionals using the Personal Health Record (the Blue Book) was informative enough [*See, I'm not too big on that… Only because you know she's kind of on the lower side and that freaks me out…Once a month is enough for me. Mother 18]*.

##### Practical Content and Delivery

The need for updated and accurate information as well as guidance around infant feeding practices was voiced by the mothers *[Absolutely, yeah, very important…Examples of main meals…some more ideas on suitable finger foods that aren't a choking risk. Mother7]*. While other mothers disagreed on receiving information about the frequency of feeding their baby *[I don't know about the when and how kind of thing, because you don't really go off–…you know your baby—when you're a mum, you know. Mother 4]*.

However, a common point emphasized from several mothers was the anxiety of receiving information that would cause the mother to feel guilty about her feeding practice *[I guess things that are going to make parents feel bad about their decisions and, I don't know, push certain ideas too much. Mother 21]*.This was voiced about milk feeding practices *[one thing you'd need to be careful about would be to make sure you know it doesn't become something on the breast—you know, they call the breast Nazis, because breast is best and nothing else will do kind of thing…it would need to be a balanced. Mother 1)*. Also first foods introduced to their baby *[if the website was offering advice etc and say if I wrote in saying I've decided to let [baby's name] have fish now, even though technically you're not supposed to give it until they're 9 months, and if somebody came back to me saying I was doing it wrong. Mother 20]*.

## Discussion

The findings of the two independent studies discussed in this paper were similar in that both pregnant women and mothers were interested in using apps for parenting support. In Study 1, the cross-sectional survey study with pregnant women, 100% of the women reported downloading an app on their phone either free or paid. Further, a higher level of education was significantly associated with likelihood of using an app for promoting healthy infant feeding practices. In the qualitative study, the mothers also held positive views of a hypothetical app that could support them with healthy infant feeding practices. In particular, the mothers indicated that features such as receiving personalized messages and videos would be beneficial to include in the app.

In both studies, a targeted aim was to explore women's use of apps and interest in an app that supported healthy infant feeding practices. The results from both studies are aligned with the existing literature indicating that women do have an interest in apps on this topic (11, 36). However, similar to our cross-sectional survey study, an Australian study also reported that women from a higher level of education were more likely to use an app for parenting purposes (11). Despite this being said, the qualitative study described in this paper and findings from other studies (37, 38) have illustrated that mothers from a low socioeconomic background also share an interest in using an app that provides support with infant feeding. The Growing healthy trial, an mHealth intervention on infant feeding, further supports this, as more than half of participants recruited had an education level lower than university (39). The studies discussed in this paper coupled with the supportive literature emphasize on the potential that mobile phones can bridge the gaps in health disparities across demographic groups (40).

The mothers in the qualitative study expressed their interest in various features they thought would be beneficial as part of an app that supports with infant feeding information. These findings are aligned with another qualitative study which identified those pregnant women and mothers value apps which are multifunctional providing them support with information and encourages social connections with other mothers (28). Further, it has been suggested that using multiple modes or features with a health program enhances the uptake of the desired behaviors (41). For instance, a website-based intervention can provide detailed information (42), while forums provide peer support to mothers (14) and personalized messages can trigger reminders for desired behaviors (43). These features differ in the mode of delivering the health information to the end user and potentially increase participation rate to influence engagement levels and uptake of desired behaviors. However, participants' engagement levels decreased when they were used as a stand-alone feature (14, 42, 43). Evidence suggests that interventions which used a combination of mHealth features have facilitated in a greater uptake of behavior change such positive changes to diet, physical activity and weight status (44) or practicing healthy household routines that are associated with the prevention of childhood obesity (45)

Direct contact with participants through messages was another feature that interested the mothers in the qualitative study. Similar findings to another study (46), there was variability on acceptable rates of contact through messaging, ranging from once a day to once a month. Several behavior change interventions determined the frequency of contact to participants based on the expected frequency of the target behavior (47, 48). Despite the benefits of this feature which has shown to improve short term behavior change, excessive contact was noted as a concern by mothers in this study and others (46). The exploration of acceptable rates of contact by interventions for behavior change is needed to prevent potential adverse effects or disengagement with the program.

Mobile health programs supporting behavior change have been identified as useful (40, 49). We found that the mothers were enthusiastic about receiving information and advice about infant feeding practices, yet they also emphasized their concern about receiving content which seemed judgmental. For instance, there are conflicting views about milk feeding behaviors, such as the “breast is best” message vs. formula feeding and also the timing of solid introduction at 4 months or at around 6 months as per recommended in the National Guidelines (23). There is research to support that if information is perceived as judgmental it can lead to increased stress impacting on their competency to adjust to their new role as a parent (1). Therefore, as interventions delivered through mobile apps can potentially cause distress it is necessary to consider this in the design and evaluation of parenting interventions. Further research needs to be conducted to explore how parents interpret information to understand what parents might consider judgmental advice.

Overall, there are several motives on why apps should be considered as a mode to deliver infant feeding information. Firstly, this paper has ascertained that although there are various sources of infant feeding information available, both expectant parents and mothers regardless of socioeconomic status expressed an interest in receiving information through an app form. Secondly despite the proliferation of health apps (17), there is a dearth of evidence-based infant feeding apps (22) and studies reporting the effectiveness of using mHealth to prevent unhealthy weight gain in infants (43, 50). Further investigation is warranted to explore the effectiveness of using apps and other technology with this demographic. Thirdly, as childhood obesity and its determinants such as high rates of early breastfeeding cessation and early introduction to solids (51) still remain a public health issue that needs to be prioritized and addressed. Lastly, the use of technology such as mobile phones and the internet will continue to rise (52) and despite the growth and changes within technological capacity, apps will likely contribute as the one of many health information sources that will be used as a service by the public.

## Strengths and Limitation

A strength of this paper is that it presented two independent studies through quantitative and qualitative research across from pregnant women to mothers to illustrate that there is an interest and a need to develop apps supporting with infant feeding. Further, the use of telephone to conduct in-depth qualitative interviews offered flexibility in interview times which allowed participation by mothers who have other obligations such as looking after their infant.

However, the cross-sectional survey was limited by its survey respondents. Selection bias was evident due to the selection criteria of the study participants. The survey was designed as baseline measures for a feasibility study of the Healthy Beginnings app which was only available for iPhone users. A limitation of the qualitative interviews was that the participants were asked questions regarding features they would like to see in a hypothetical app which they had never seen or used. Further larger scale studies are required to confirm these findings. Despite the small sample size, the similarity of findings with other qualitative studies (12, 46) exploring mothers' perspectives on mHealth interventions such as using multiple technology features and practical information and delivery suggest that these are common interests.

## Conclusion

The two independent studies described in this paper found positive perspectives regarding the development of an app that would provide support to mothers with infant feeding practices. Our findings suggest that using apps or other technology is an acceptable mode to deliver information to this demographic regardless of their socioeconomic status. This is particularly important as there are existing health disparities with people from a low sociodemographic background compared with their counterparts. In particular participants expressed an interest in the use of various features to be part of an app delivering infant feeding information which is an important element of engagement. This research contributes to growing literature in identifying the opportunities to build engaging mobile apps and also the barriers which may inhibit the uptake of these interventions from this demographic. There is great potential for health promotion practitioners to be engaged in app development for the purpose of promoting health in early years and health promotion in general.

## Author Contributions

LMW and HX worked on Study 1, the cross-sectional study. This included the development, implementation and analysis of the cross-sectional survey study. ST, CGR, RAL, KC, and ED-W worked on Study 2, the qualitative interviews. ST and CGR worked closely on the development, implementation and analysis of the qualitative study. RAL, KC, and ED-W assisted with analysis of the qualitative study and the writing of the paper.

### Conflict of Interest Statement

The authors declare that the research was conducted in the absence of any commercial or financial relationships that could be construed as a potential conflict of interest.
